# Improving Development of Drug Treatments for Pregnant Women and the Fetus

**DOI:** 10.1007/s43441-022-00433-w

**Published:** 2022-07-25

**Authors:** Anna L. David, Homa Ahmadzia, Richard Ashcroft, Christina Bucci-Rechtweg, Rebecca N. Spencer, Steve Thornton

**Affiliations:** 1 Elizabeth Garrett Anderson Institute for Women’s Health, University College London, 74 Huntley Street, London, UK; 2 NIHR UCLH Biomedical Research Centre, 149 Tottenham Court Road, London, UK; 3 Division of Maternal-Fetal Medicine, Department of Obstetrics and Gynecology, The George Washington University, Washington, DC USA; 4 City University, London, UK; 5 Head, Pediatric and Maternal Health Policy, Global Drug Development Regulatory Affairs, Pediatric Center of Excellence, Novartis Pharmaceuticals Corporation, Basel, Switzerland; 6 University of Leeds, Leeds, LS2 9JT UK; 7 Queen Mary University of London, Dean Rees House, 2nd Floor, Charterhouse Square, London, EC1M 6BQ UK

**Keywords:** Pregnancy clinical trial, Fetus, Adverse event, Safety, Therapeutics

## Abstract

The exclusion of pregnant populations, women of reproductive age, and the fetus from clinical trials of therapeutics is a major global public health issue. It is also a problem of inequity in medicines development, as pregnancy is a protected characteristic. The current regulatory requirements for drugs in pregnancy are being analyzed by a number of agencies worldwide. There has been considerable investment in developing expertise in pregnancy clinical trials (for the pregnant person and the fetus) such as the Obstetric-Fetal Pharmacology Research Centers funded by the National Institute of Child Health and Human Development. Progress has also been made in how to define and grade clinical trial safety in pregnant women, the fetus, and neonate. Innovative methods to model human pregnancy physiology and pharmacology using computer simulations are also gaining interest. Novel ways to assess fetal well-being and placental function using magnetic resonance imaging, computerized cardiotocography, serum circulating fetoplacental proteins, and mRNA may permit better assessment of the safety and efficacy of interventions in the mother and fetus. The core outcomes in women’s and newborn health initiative is facilitating the consistent reporting of data from pregnancy trials. Electronic medical records integrated with pharmacy services should improve the strength of pharmacoepidemiologic and pharmacovigilance studies. Incentives such as investigational plans and orphan disease designation have been taken up for obstetric, fetal, and neonatal diseases. This review describes the progress that is being made to better understand the extent of the problem and to develop applicable solutions.

## Introduction

Pregnancy is a wonderful time for most women, but some suffer from anxiety, uncertainty, and fear. Millions of women and children die each year during pregnancy and childbirth from preterm birth, fetal growth restriction (FGR), pre-eclampsia and hemorrhage. Globally, preterm birth is the second leading cause of childhood death < 5 years of age, and affects 1 in 10 infants in the US.

Increasingly women have health issues before they conceive and optimizing their pregnancies using effective pharmaceuticals is challenging. Traditionally, pregnant women have been excluded from participating in clinical trials of therapeutics. They are considered to be a “vulnerable” population in research due to their developing fetus [1]. There is no legal or regulatory requirement for new drugs to be tested on pregnant women, leading to > 80% of pregnant patients routinely receiving therapies that have not been adequately studied in pregnancy [2]. This attempt to protect pregnant women commonly leaves them reliant on efficacy data generated in non-pregnant populations and safety data from post-marketing surveillance studies. Clinicians and patients are often unaware of this evidence gap about the drugs that they are prescribing or ingesting [3]. Some have even proposed a moral imperative to include pregnant women in therapeutic trials [4]. This could provide women with informed use of effective treatments, promote fetal safety, reduce avoidable harm from suboptimal care, and enhance equitable access to potential benefits of research participation.

Pregnant women were routinely excluded from COVID-19 clinical trials, perhaps due to the initial lack of evidence about the effects of this infection in pregnancy [5]. For many trials, exclusion was not well justified as treatments being evaluated had no or low safety concerns during pregnancy. Data that emerged from multiple observational cohort studies showed that pregnant women have higher rates of severe COVID-19 infection than non-pregnant women and increased risk of pregnancy complications including preterm birth and pre-eclampsia [6]. This has resulted in higher rates of intensive care admissions, mechanical ventilation and death, with pregnant women with co-existing morbidities at even greater risk [7]. Testing of COVID-19 vaccines initially excluded pregnant participants. The more limited safety data in pregnancy explains why uptake of COVID-19 vaccines in pregnant and lactating women has been low despite active communication campaigns. As a result, pregnant women are seen as one of the populations most vulnerable to COVID-19 infections.

There has been significant progress made over the last few years in the field of pregnancy therapeutics. Initiatives such as the Task Force on Research Specific to Pregnant Women and Lactating Women (PRGLAC) has identified and addressed gaps in knowledge and research on safe and effective therapies for pregnant/lactating women and the fetus [8]. This review describes the current regulatory requirements for drugs in pregnancy and explores the barriers and potential solutions to addressing inequalities in medicines development for pregnant/lactating women and the fetus. Some progress has been made in how to assess clinical trial safety in pregnant women, the fetus, and neonate and how to refine trial inclusion and exclusion criteria. There are also innovative methods to model human pregnancy pharmacology using computer simulations and novel ways to assess fetal well-being and placental function. Governments, regulators, researchers, women and their families, and the pharmaceutical industry are engaging to identify methodologies that will facilitate generation of data to better inform medicines use in pregnancy. These initiatives will provide women access to information that they deserve about the medicines they are using.

## Current Issues

### The Requirement to Investigate Drugs in Pregnant Populations

The COVID-19 pandemic has focused attention on the harm to pregnant populations who are excluded from investigative drug trials [9]. Initially pregnant populations were excluded from all of the COVID-19 vaccination trials so that when vaccination was initially rolled out, pregnant women were not called forward because there was uncertainty about whether the vaccines were safe and effective in pregnancy. This resulted in major harm with increased rates of preterm birth, stillbirth and neonatal morbidity in unvaccinated pregnant women [10, 11]. In the UK, a Health England report in October 2021 showed that one in five of the most critically ill Covid-19 patients in hospital were unvaccinated pregnant women [12]. It is now clear that babies born to women who were vaccinated during pregnancy, particularly during the third trimester, benefitted from transplacental transfer of maternal antibodies. In addition babies were 61% less likely to be admitted to hospital with Covid-19 in the first six months [13].

It is surprising that drug development in pregnancy has been neglected, since improving fetal outcome could be a major benefit with significant social and financial consequences. The risk of long-term adverse consequences may have led to a reticence to investigate and develop treatments which could improve the life of so many. Well publicized drugs used in pregnancy such as thalidomide and diethylstilbestrol were initially considered used without adequate safety assessment and were only subsequently demonstrated to have significant effects in the offspring of mothers taking them. This has led to a widespread reluctance from many sources to promote the development of drugs that women deserve, to ameliorate mortality and long-term morbidity.

There are three major reasons to develop and investigate drugs in pregnant/lactating populations and the fetus. First, many pregnant women choose not to take existing drugs unless they can be assured of safety since the required clinical data is not always readily available. Often the safety data have been accumulated over many years meaning that pregnant women are given outdated therapies rather than newer drugs that have not been adequately tested [14]. Furthermore, there is little incentive for commercial organizations to license drugs in pregnancy given the high costs, the potential for long-term harm, and limited financial benefits.

The second reason is to improve pregnancy-specific conditions such as pre-eclampsia, preterm labor, and FGR. Although the number of women treated and treatment duration is relatively small, the potential for long-term benefit is significant. Preventing a lifelong condition such as cerebral palsy would have tremendous personal, social, and economic advantages. Although there may be little commercial drive to develop new drugs for these indications, there is considerable interest due to the financial consequences of long-term morbidity. Investigational drugs designed to treat pre-eclampsia, preterm labor, and/or FGR have even received orphan disease designation, bringing reductions in the cost of scientific advice and advantageous protection from market competition [15].

The final reason is the increasingly common diagnosis of life-threatening congenital and rare fetal diseases, where intervention in pregnancy may improve neonatal and long-term outcomes. Opportunities for non-invasive prenatal diagnosis using circulating fetal DNA and the expansion of genetic diagnosis through new genomic technologies is allowing parents to receive a definitive diagnosis of a serious congenital disorder before birth, making the option of fetal therapy a reality. Examples of recent clinical trials of fetal therapy include in utero stem cell transplantation for alpha major thalassaemia and osteogenesis imperfecta [16], and intra-amniotic protein injection for X-linked hypohidrotic ectodermal dysplasia [17].

An important consideration in developing investigational drugs for pregnancy-specific conditions are the difficulties in conducting clinical trials. Many of the conditions that affect the mother and fetus such as pre-eclampsia, fetal growth restriction and congenital fetal anomalies are uncommon and are even defined as rare diseases with orphan designation [15]. This is compounded by public and professional views of research in pregnancy and the need to consider the effects of drug administration on both the mother and fetus. Such issues increase clinical trial complexity and cost and make trials difficult to complete. It is clear that affected families welcome the chance to explore potential treatments for pregnancy-specific maternal and fetal diseases [18–20]. In the face of poor fetal outcome, clinical trials of investigational drugs for pregnancy-specific diseases can recruit well. Two STRIDER clinical trials of sildenafil citrate for early onset FGR recruited well and completed enrollment on time [21, 22]. Disease severity was high with around one-third of pregnancies affected by a perinatal loss. In this situation, parents welcomed the opportunity to participate in clinical trials [23].

Although women are prepared to be involved in trials, there may be difficulties satisfying regulatory requirements. In a recent clinical trial of a new drug for preterm labor, standard of care was to give a tocolytic to delay delivery in threatened preterm labor. However, improved outcome had never been conclusively demonstrated with tocolytic therapy [24]. The regulatory requirements for the trial were complex due to the need to demonstrate improvements in both maternal and fetal outcomes, including long-term follow up of the offspring. Placebo controlled studies were required and ethics review boards were reticent to approve a placebo-controlled trial when early delivery could have a devastating effect on the infant. Clinicians were similarly uneasy about recruiting to the trial as they considered initial short-term tocolysis to be standard of care. However, with no evidence to support the standard of care, clinical equipoise between the unsubstantiated standard of care, placebo and the treatment under development existed. In addition, with limited evidence to support the standard of care, it would have been difficult to assess the clinical meaningfulness of the new treatment relative to what is standard of care. Coupled with the complexities of a multifactorial condition and the need to recruit a specific gestational age range, it is not surprising that recruitment was low leading the trial to be discontinued.

There is also resistance from Higher Education Institutions and healthcare providers to support pregnancy-related research, often citing financial or legal barriers, particularly related to insurance costs. There remains a widespread lack of understanding of fetal physiology/development and the potential impact of drugs at different gestational ages needs to be clearly communicated by clinicians.

Nevertheless, there is real hope that the current situation is changing. A number of organizations in the US, EU, and UK are driving the need to develop therapeutic agents in pregnant and lactating populations. Women can choose whether to be involved in trials during pregnancy and there are discussions with governments about incentivizing pharmaceutical companies to license drugs for use in pregnancy. Such changes will enable the development of new drugs and also address the use of unlicensed drugs that are commonly recommended in national guidelines. This will increase the likelihood that licensed drugs are developed for use in pregnant/lactating women and the fetus and are subject to the rigorous efficacy and safety evaluations required for licensing.

### Current Regulatory Requirements for Drugs in Pregnancy

Effective regulation of medicines development is required to ensure the safety and efficacy of drugs for use in the general public. Most governments have established regulatory agencies which publish guidance that reflects current thinking on trial safety that industry should consider when developing drugs. The International Council for Harmonisation of Technical Requirements for Pharmaceuticals for Human Use (ICH) brings together regional regulatory agencies and the pharmaceutical industry to discuss all aspects of drug registration. As part of the ICH scientific consensus process, guidelines are developed to achieve greater harmonization in global drug development processes. Membership in the ICH requires regional regulatory agencies and manufacturers to adopt the output of consensus proceedings into regional regulatory and development practice. Familiarity with these guidelines is inherent to understanding why pre-licensure clinical trials of investigational drugs often exclude pregnant populations and/or require contraception in women of childbearing potential (WOCBP).

When evaluating investigational drugs in WOCBP, the ICH community has a high level of concern for the unintentional exposure of a fetus before information on the potential benefits versus risks is available. As a result, ICH regions have adopted similar recommendations related to the type and timing of non-clinical reproductive toxicity studies required to support the inclusion of WOCBP in clinical trials (*ICH S5 R3; ICH M3 R2*) [25, 26]. The ICH M3 R2 highlights the importance of characterizing and minimizing risk of unintentional exposure of a fetus when including WOCBP in clinical trials. These approaches include the conduct of reproductive toxicity studies to characterize the inherent risk of a drug while developing appropriate precautions during exposure of WOCBP to investigational drugs in clinical trials. Risk can also be limited by preventing pregnancy during clinical trials using pregnancy testing (e.g., β-subunit of HCG), highly effective methods of birth control, and/or allowing study entry only after a WOCBP has a confirmed menstrual period.

ICH guidance also outlines specific circumstances where WOCBP could be considered for inclusion in early clinical trials prior to the completion of non-clinical developmental toxicity studies (*ICH S5 R3; ICH M3 R2*) [9, 10]. This is particularly important when a disease occurs predominantly in women, when the objectives of the clinical trial cannot be met effectively without inclusion of WOCBP, and where sufficient precautions to prevent pregnancy have been incorporated.

The ICH regulatory regions require the full complement of female reproductive toxicity studies (*ICH S5 R3; ICH S6*) [25, 27] and genotoxicity tests (*ICH S2 R1; ICH S9*) [28, 29] to be completed before inclusion in any clinical trial of WOCBP not using highly effective birth control or whose pregnancy status is unknown (*ICH M3 R2*) [10]. Similarly, ICH guidelines require female reproductive toxicity studies and genotoxicity tests to be conducted and safety data from previous human exposure to be evaluated before the inclusion of pregnant women in clinical trials for investigational drugs (*ICH M3 R2*) [10].

Regulatory agencies expect that medications approved for use in pregnant or lactating women must first be shown to be safe and effective. In contrast, when medications are approved to treat general medical conditions that can occur in pregnant women (e.g., seizures), it is common for safety and effectiveness data that defines the labeled use of the drug to be collected solely from non-pregnant or non-lactating adults. Regulatory labels often indicate that comprehensive dosing, pharmacokinetic, and pharmacodynamic information of many drugs used in pregnancy and lactation are inadequate or unavailable. Labeling may also only include non-clinical data relevant to pregnancy and lactation, which can be difficult for clinicians and patients to interpret as the clinical relevance of these findings are largely unknown. In addition, vaccines and medicines taken during pregnancy are particularly susceptible to product liability litigation. Together these barriers routinely delay the acquisition of information about the use of a drug in pregnancy and lactation until safety and effectiveness have been established in non-pregnant disease populations (i.e., after product licensure). As physiologic changes occur in women during pregnancy, there is a pressing need to identify earlier approaches to generate data in pregnant and lactating women in clinical studies.

With the exclusion of drugs and biological products developed to treat conditions unique to pregnancy, there are often no or limited human data to inform the safety of the drug taken during pregnancy at the time of a drug’s licensure. In spite of this, drugs labeled for use in adults are also approved for use in pregnant women barring a Contraindication statement for their use during pregnancy in the drug label. Therefore, the use of these medicines in pregnancy for concomitant adult diseases is not considered “off-label”. Although this does not erase the knowledge gap, it further underscores the need to identify solutions to support more informed use of medicines in pregnancy.

The US Food and Drug Administration (FDA) has recently issued draft guidance entitled ‘Pregnant Women: Scientific and Ethical Considerations for Inclusion in Clinical Trials’ [30]. The guidance is intended to facilitate discussion across stakeholder groups to promote maternal and fetal health and inform prescribing decisions during pregnancy. The agency acknowledges the challenges of including pregnant women in drug development research, outlines the interdependencies of maternal and fetal well-being, and provides recommendations for inclusion of pregnant populations based on ethical principles and clinical need. The draft guidance also calls for the “judicious inclusion of pregnant women in clinical trials and careful attention to potential fetal risk” and encourages sponsors to meet with the relevant review divisions to discuss when and how to include pregnant women in drug development. An important new recommendation to sponsors in this guidance is the proposal that women who become pregnant while enrolled in a clinical trial be allowed to continue “on an investigational drug if the potential benefits of continued treatment outweighs the risks of ongoing fetal exposure”. This recommendation offers a meaningful opportunity to sponsors to incorporate protocol-defined prospective data collection during pregnancy to inform dosing, effectiveness, and safety in pregnancy. At present, this is the only such guidance across ICH regions addressing the inclusion of pregnant populations in investigational research trials.

### Clinical Trial Expertise in Pregnant/Lactating Populations

The Maternal–Fetal Medicine Units (MFMU) Network was established in 1986 by the National Institutes of Child Health and Human Development. It includes 12 clinical centers and a data coordinating center that conduct clinical trials in obstetrics. The major aims of the MFMU Network are to: (1) reduce the rates of preterm birth, FGR, neonatal morbidity, and maternal complications of pregnancy, and (2) evaluate maternal and fetal interventions for efficacy, safety, and cost-effectiveness. The Network has a total of 54 ongoing or completed studies (31 randomized trials, 23 observational studies) [31]. Some of the landmark therapies based on the results from these trials include antibiotics for prolongation of latency in the setting of preterm premature rupture of membranes (PPROM), betamethasone for late preterm lung maturity, and maternal magnesium sulfate for neonatal neuroprotection.

The Obstetric-Fetal Pharmacology Research Centers Network also supports translational research to improve the safety and effective use of therapeutic drugs during pregnancy and lactation [32]. It currently has three centers in the US and through 2020 had published 194 peer-reviewed publications on pre-eclampsia, gestational diabetes, preterm birth, mental health disorders, and many others [32]. The overall goal of the network is to promote and facilitate cooperative multidisciplinary research that is focused on obstetric pharmacokinetics and pharmacodynamics. Other initiatives to promote pregnancy clinical trials include workshops such as the MHRA/Gates sponsored training day on “Pharmacokinetics of medicines in pregnancy—understanding how pregnancy affects plasma drug levels” [33].

### Reproductive Toxicology Considerations

There are many anatomical and physiologic alterations in pregnancy that alter the way drugs are absorbed, distributed, metabolized, excreted, and transported [34]. For example, progesterone causes slowing of gastrointestinal motility and absorption can be delayed. Increases in blood volume and weight gain are associated with increased distribution. In terms of safety, consideration of both the pregnant woman and fetus is necessary. Pharmacokinetics and pharmacodynamics studies may be necessary to optimize the use of drugs already prescribed in pregnancy or when studying new drugs designed for pregnancy-specific conditions [35]. Investigational drugs in particular need pre-clinical studies to demonstrate safety prior to clinical use. Resources like Reprotox and Lactmed compile animal and human data for risk of malformations or toxicity and are often used by clinicians to counsel pregnant women [36, 37]^.^ However, limitations with these resources include the maternal comorbid conditions for which the drugs are used and any pathological consequences for the pregnancy. For example, labetalol use has been linked to FGR but it is medically indicated to treat chronic hypertension or pre-eclampsia, which is also associated with FGR. This challenge of linking these drug associations is compounded by polypharmacy, any maternal comorbidities that may pre-exist, and pregnancy-related diseases such as pre-eclampsia that will affect drug absorption, metabolism, and excretion.

### Global Concerns

Conduct of pharmacologic studies in pregnant women on a global scale does not always translate into global changes in clinical care. The London School of Hygiene and Tropical Medicine Clinical Trials Unit conducted several studies of pharmacologic interventions in pregnant women to reduce morbidity and mortality associated with postpartum hemorrhage [38–40]. These studies are primarily being conducted in low- and middle-income countries (LMIC) given that this is where 99% of maternal mortality from hemorrhage at delivery occurs [41]. However, clinical guidelines often lag behind clinical trials until multiple randomized trials demonstrate the same findings. The argument often used in high resource settings is that the study is conducted in low resource settings and the findings may not necessarily be generalizable. This presents an ethical dilemma of how many clinical trials are considered sufficient to change clinical care worldwide. A balance is needed between the risks of missing an adverse outcome due to insufficient numbers of trial participants versus the potential ongoing harm through delays in implementing effective drug treatments. Furthermore, who is qualified to make the determination that a certain drug finally merits routine use in all clinical care settings, irrespective of resource availability? It is also important to seek input from healthcare providers and regulatory authorities as well as the women who are active participants in their care. That may not be easy to achieve in low resource settings where patient participation may not be embedded in the clinical research paradigm. Decisions about whether to start or stop clinical trials must take into consideration multiple points of view.

Given the numerous challenges described, providing evidence-based treatments for pregnant women and their fetuses may not be an achievable goal. However, much work has been done over the last few decades to address issues arising in trial design, participant selection, safety monitoring, and outcome reporting which is likely to be transformational. In addition, innovations in non-invasive fetal and placental functional assessments will make it easier to measure pregnancy well-being and the potential effects of drug interventions.

### Better Communication About Safety: Maternal and Fetal Adverse Event (AE) Consensus

Conducting clinical trials in pregnancy raises many challenges, primarily due to safety concerns for the mother and fetus, particularly when testing novel maternal and fetal therapies. The paucity of clinical trials in pregnancy has led to absent standard frameworks such as standardized severity grading for maternal and fetal AEs. This renders clinical trials in pregnancy more difficult and can compromise the health of pregnant participants.

Although they may not necessarily have a causal relationship with the investigational drug, AEs are important signals in clinical trials, facilitating swift and responsible communication of safety data between study investigators, sponsors, and regulators [42, 43]^.^ AEs should be recorded in medical records and reported to the sponsor and other relevant authorities. A decision should then be made as to whether they meet the regulatory definition of ‘serious’ and are directly related to the administration of the investigational drug. This will determine whether to classify the event as a serious adverse reaction (SAR). AE severity is recorded using standard grading criteria, commonly the common terminology criteria for adverse events (CTCAE) (Version 5.0) which comprises 837 potential AEs [44]. Grading AEs allows decisions around dose escalation to be rendered more objectively and also permits comparison of AEs between clinical trials. The CTCAE contains AEs related to ‘pregnancy, the puerperium, and perinatal conditions’ including fetal death and/or growth retardation, premature delivery, pregnancy, puerperium, and other postnatal conditions. Some condition-specific severity grading for pregnancy-specific events have been developed (e.g., HIV-AIDS, surgery) [45, 46]. However, until recently there were no standard general severity grading criteria. This contrasts with Delphi consensus work to integrate neonatal terminology and definitions into wider dictionaries undertaken by the International Neonatal Consortium [47, 48]. The Neonatal Adverse Events Severity Scale version 1.0 classifies neonatal AEs into 5 grades (mild, moderate, severe, life threatening, or death) with severity defined by the effect of the AE on age-appropriate behavior, basal physiologic functions, and healthcare changes in response to the AE.

Through an international Delphi consensus process involving healthcare professionals and patient groups, a team has systematically developed definitions and severity grading for maternal and fetal AEs called MFAET Version 1.0 [49]. Fetal AEs had to be diagnosed in utero, with the potential of severe AEs to cause a detrimental effect before birth. New fetal AE definitions were developed by considering the different organ systems that might be affected and were adopted by the Medical Dictionary for Regulatory Activities (MedDRA) in 2016 [50]. A generic fetal grading system was based on CTCAE criteria and then AE severity was graded independently for the pregnant woman and fetus (Table 1). These 12 new maternal and 19 fetal AE definitions and severity grading criteria were then ratified by consensus. This terminology is available at MFAET version 1.0, fills a vital gap in maternal and fetal translational medicine research, and supports the development of therapies for pregnant women and their neonates [51].

**Table 1:** MFAET v1.0.

Maternal AEs	Fetal AEs
Hemorrhage in pregnancy	Hemorrhage in pregnancy
Preterm premature rupture of membranes	Preterm premature rupture of membranes
Chorioamnionitis	Chorioamnionitis
Anemia of pregnancy	Anemia of pregnancy
Gestational hypertension	Fetal fluid collection^a^
Pre-eclampsia	Fetal bradycardia: non-labor^a^
Eclampsia	Fetal tachyarrhythmia^a^
Premature labor	Cardiac function abnormalities^a^
Puerperal infection	Fetal brain scan abnormal^a^
Postpartum hemorrhage (primary)	Fetal gastrointestinal tract imaging abnormal^a^
Retained placenta or membranes	Fetal musculoskeletal imaging abnormal^a^
Amniotic fluid embolism	Fetal renal imaging abnormal^a^
	Fetal movement disorders^a^
	Fetal neoplasm^a^
	Fetal structural abnormalities: not otherwise classified^a^
	Abnormal fetal growth^a^
	Fetal intraoperative injury^a^
	Procedural hemorrhage^a^
	Post-procedural hemorrhage^a^

The maternal and fetal AE terms for which definitions and severity grading criteria were developed [49]

^a^Added to the Medical Dictionary for Regulatory Activities terms list

### Developments in Trial Design

As few drug trials are conducted during pregnancy and lactation, improvements in trial design may not necessarily be applicable. For early phase trials where drugs are being used for the first time in humans, dose escalation studies are usually performed [52]. Classical dose escalation has used a rule-based design such as a 3 + 3 design with cohorts of three patients studied at pre-specified dose levels to reach the maximum tolerated dose (MTD). However, over the last thirty years developments in adaptive trial design in oncology, such as the continual reassessment method (CRM), have allowed for more flexibility with faster acceleration to a potentially therapeutic dose [53]. This method uses a statistical Bayesian stepwise approach to integrate the accumulated observed data in the trial with prior information from clinicians and past studies. It then provides a recommendation as to the dose for the next cohort or patient in the trial. This means that early phase clinical trials can be carried out more efficiently by escalating through lower doses, so that fewer participants receive a sub-therapeutic dose. Unfortunately, these advances have yet to benefit pregnant trial participants. This may be due to a host of influences, though the outsized effect results from the lack of inclusion of pregnant populations in drug development trials which limits the ability to utilize such methodologies. However, such trial designs could improve the balance of risks and benefits in early phase trials.

### Developments in Drug Delivery During Pregnancy

Delivering drugs for maternal indications is unlikely to require the introduction of any novel techniques. However, this is not the case for the placenta or fetus, compartments that need to be reached via the mother and for which more targeted therapies may be required. Examples include fetal intravascular injection accessed using ultrasound guided imaging via the umbilical vein. This is currently the route of delivery chosen in two trials of in utero stem cell transplantation [16]. The placenta could be reached for drug delivery via direct intraplacental injection (similar to chorionic villus sampling) or via the uterine artery using interventional radiology techniques [54]. However, both of these techniques are invasive and may compromise the pregnancy. Research is now focusing on specific placental targeting techniques delivered into the maternal circulation. This includes non-viral polymers that are capable of delivering plasmids, small interfering RNA, and other effector nucleic acids to the dysfunctional placenta, similar to cancer therapeutics [55].

### Improving Assessment of the Fetus and Placenta

Assessing the impact of an intervention on a pregnant trial participant involves both the mother and fetus. Routine application of the Amsterdam consensus criteria to sample the placental and analyze histologically allows international comparability of clinicopathologic studies of the placenta in clinical trials [49]. Examining the direct effect of an intervention on the fetus and the placenta is more challenging. However, there are promising developments in fetal heartbeat and movement monitoring, fetal and placental imaging, and markers for fetal and placental well-being in maternal blood that may improve safety and efficacy monitoring in the future.

Cardiotocography (CTG) or a non-stress test, the external electronic detection of the fetal heartbeat and uterine activity via maternal abdominal monitors, has been used clinically to assess fetal well-being since the 1970s. Computerized analysis of the antenatal CTG (cCTG) via the application of objective Dawes-Redman criteria is increasingly used in clinical practice, particularly in the setting of high-risk pregnancies [56, 57]. For example, cCTG was one of the three fetal monitoring arms of the TRUFFLE clinical trial that investigated the optimum decision tool for delivery in the presence of early onset FGR [58]. cCTG analysis includes measurements of short-term variability which may more reliably detect fetal hypoxia than traditional CTG analysis. Fetal ECG monitoring has also been applied in labor to detect intrapartum fetal hypoxia with limited success [59]. On-going work using machine learning is being applied to both the intrapartum CTG and ECG which may lead to more reliable assessment of fetal well-being in the future [60].

Other indicators of fetal well-being include fetal body and breathing movements. These have previously been assessed as part of the biophysical profile, a relatively labor-intensive procedure involving up to 30 min of ultrasound observation [61, 62]. Advances in machine learning are leading to the automation of these assessments and the development of wearables to allow longer-term monitoring [63, 64]. Such technology could improve assessment of the short- and medium-term fetal response to interventions.

Assessment of fetal size, structure, and fetoplacental circulation using ultrasound is the mainstay of antenatal care in high resource settings and increasingly in LMIC with the advent of less expensive portable scanners. Where available, magnetic resonance imaging (MRI) provides a useful clinical adjunct for assessing structural fetal anomalies particularly in the central nervous system [65]. Deep learning strategies are now being applied to ultrasound and MRI imaging of the fetus and placenta, providing new assessment methods through techniques including classification, segmentation, object detection, and tracking [66].

One promising area is MRI measurement of oxygenation within the maternal and fetal placental compartments and fetal circulation with further developments expected to come from the NICHD Human Placenta Project [67–69]. Although such measurements would be especially important as indicators of efficacy in trials of therapies for FGR, they could also provide safety signals in trials of therapeutics that have the potential to affect uterine perfusion. Novel techniques to evaluate fetal neurodevelopment using four-dimensional ultrasound assessment of fetal movement may provide an early signal of fetal neurological deficits and fetal pain, an important consideration in trials involving interventions targeting the fetus [70, 71]. The importance of fetal analgesia and anesthesia for interventions is highlighted by the recently updated Society for Maternal-Fetal Medicine guidelines for fetal surgery [72].

Non-invasive prenatal diagnosis of fetal aneuploidy, single gene disorders, and blood group through the analysis of circulating DNA in maternal blood samples is well established in clinical practice. Placentally-produced proteins can provide surrogate markers of placental function and/or damage, such as lower maternal serum concentrations of placental growth factors in placental insufficiency and pre-eclampsia [73]. Study of circulating mRNA and miRNA markers may allow for a more detailed assessment of placental gene expression and function while providing non-invasive indicators of fetal hypoxia [74]. Factors such as maternal body mass index and the maternal contribution to products that are not placenta specific are limitations of measuring circulating proteins and RNA in maternal blood. A more targeted assessment of the placenta may be possible by analyzing the cargo of placental extra-cellular vesicles, lipid-bound structures containing proteins and RNA from their tissue of origin which could act as a ‘liquid biopsy’ of the placenta [75].

### Improving the Reporting of Trials in Pregnancy

As pregnancy, childbirth, and subsequent lactation covers many healthcare practitioners and the transition from a pregnant patient to a mother and neonate, it has been difficult to analyze all the important outcomes. These need to include data from multiple sources including primary, secondary and tertiary care as well as from midwifery, obstetric, fetal medicine, and neonatology perspectives. In 2014 the core outcomes in women’s and newborn health (CROWN) initiative was launched to address the huge variability in reported outcomes of previous clinical trials in pregnancy [76]. Among the core outcome sets now available are those for trials aimed at specific pregnancy conditions such as prevention of preterm birth, treatment of pre-eclampsia, and trials to assess the effectiveness of pre-pregnancy care for existing medical conditions in pregnancy [75, 77–81]. Core outcome sets are also under development or being planned for a further 39 conditions or interventions [82, 83]. Core outcome sets not only improve the quality of individual clinical trials but also maximize their utility by allowing easier comparison of outcomes between trials and easier synthesis into meta-analysis.

### Enhancing Pharmaceutical Design and Pharmacovigilance

Machine learning (algorithms such as random forest, support vector machines, K nearest neighbors, Naive Bayes, deep learning) is a form of artificial intelligence that is increasingly being applied into research of drug development and toxicity profiling [84]. Machine learning, quantitative models, and other tools that analyze and integrate data can also be particularly useful when dealing with rare diseases in pregnant women and the fetus. Modeling using in vitro advanced technologies enhances our ability to understand interactions between different compartments when studying the pharmacokinetics and pharmacodynamics of a drug [85]. The role of the placenta in drug transfer is complex [86]. The gestational diabetes drug glyburide has been used as a model compound in a specific application of an in vitro model to better understand placental transfer [87]. Using a microengineered human placental barrier, researchers were able to demonstrate that the system was capable of mediating efflux transporter-mediated active transport that mimicked the known limited placental transfer of this maternally administered drug.

Physiologically-based pharmacokinetic modeling (PBPK) is also gaining interest and new software originally developed for use in pre-clinical models has been refined to extrapolate to humans or between human populations [88]. A variety of PBPK model suites are available based on the three original models that have been developed as part of Simcyp [89], GastroPlus [90], and OSP software suites [91]. Models need to be able to handle the significant complexity associated with pregnancy. In addition to the standard compartments of non-pregnant women, they also need to consider how and whether to incorporate supplementary compartments that are either specific to pregnancy or are of specific relevance during pregnancy. Although altered drug absorption in pregnancy and the ability to model proteins and large molecules makes some of these analyses more complex [88], there has been tremendous progress in pregnancy PBPK models which is likely to improve relevant drug assessments.

From a clinical perspective, tools to integrate drug dosing into the electronic medical record have been shown to reduce medical error and improve safety [92]. As electronic medical records continue to be refined and integrated with pharmacy services in both inpatient and outpatient settings, this will likely improve the strength of pharmacoepidemiologic and pharmacovigilance studies. Despite recent advances, the field of pharmacometrics has incorporated pregnancy as a unique area that needs further study [93].

The lack of data to inform product labels negatively impacts decision-making related to drug use during pregnancy. Numerous stakeholder groups have been working collaboratively to raise awareness of this knowledge gap and offer solutions (see Table 2). These initiatives have addressed a range of topics that impact drug research in pregnant women including impediments to investments in innovation to scientific, ethical and legal challenges. The varied participants whose perspectives have been represented have offered a diversity of solutions including requiring trial sponsors to provide scientific justification when excluding pregnant populations in studies and the opportunity for women who conceive while participating in a trial to be given an informed option to remain in the study to collect relevant PK, safety, and efficacy data. Both of these recommendations can be instituted immediately by companies, regulatory agencies, ethics boards, and individual investigators working collaboratively to ensure their incorporation.

**Table 2:** Stakeholder efforts to advance research in pregnant and lactating populations

Obstetric-Fetal Pharmacology Research Centers (OPRC) Network	Project	Obstetric-Fetal Pharmacology Research Centers (OPRC) Network|NICHD—Eunice Kennedy Shriver National Institute of Child Health and Human Development (nih.gov)
U.S. Health and Human Services (HHS) task force on research specific to pregnant women and lactating women (PRGLAC)	PRGLAC Report to the HHS Secretary and Congress, *September 2018* [8] Implementation plan, *August 2020* [94]	https://www.nichd.nih.gov/about/advisory/PRGLAC • Identified gaps in knowledge and research related to pregnant and lactating women • Provided 15 recommendations to improve therapeutic development for pregnant and lactating women • Provided an interrelated set of steps for each of the 15 recommendations
Innovative medicines initiative (IMI) conception	Project	https://www.imi-conception.eu/ • 8 work packages • To establish a system to efficiently, systematically, and ethically generate reliable evidence-based information on drug use in pregnant and lactating women • To generate, catalog, link, collect and analyze data from pharmacovigilance, modeling, healthcare, and breast milk samples
U.S. Food and Drug Administration (FDA)	Workshop New guidance documents	• Evaluation of the safety of drugs and biological products used during lactation, *April 2016* • Pregnant women: scientific and ethical considerations for inclusion in clinical trials, *April 2018* • https://www.fda.gov/regulatory-information/search-fda-guidance-documents/pregnant-women-scientific-and-ethical-considerations-inclusion-clinical-trials • Postapproval pregnancy safety studies, *May 2019* • https://www.fda.gov/regulatory-information/search-fda-guidance-documents/postapproval-pregnancy-safety-studies-guidance-industry • Clinical lactation studies: considerations for study design, *May 2019* • https://www.fda.gov/regulatory-information/search-fda-guidance-documents/clinical-lactation-studies-considerations-study-design
Duke-Margolis Center for Health Policy in cooperation with U.S. FDA	Workshop	https://healthpolicy.duke.edu/events/scientific-and-ethical-considerations-inclusion-pregnant-women-clinical-trials • Scientific and ethical considerations related to the inclusion of pregnant people in clinical trials, *February 2021*
European Medicines Agency (EMA)	Workshop Guideline	• Workshop on benefit-risk of medicines used during pregnancy and breastfeeding, *September 2020* • https://www.ema.europa.eu/en/events/workshop-benefit-risk-medicines-used-during-pregnancy-breastfeeding • Strategic reflection: EMA regulatory science to 2025 •https://www.ema.europa.eu/en/documents/regulatory-procedural-guideline/ema-regulatory-science-2025-strategic-reflection_en.pdf
Heads of Medicines Agencies (HMA)	Guideline	Guideline on good pharmacovigilance practices: pregnant and breastfeeding women, *December 2019* https://www.ema.europa.eu/en/documents/scientific-guideline/draft-guideline-good-pharmacovigilance-practices-product-population-specific-considerations-iii_en.pdf
UK Commission on Human Medicines	Report	Report of the Commission on Human Medicines’ Expert Working Group on Hormone Pregnancy Tests, *November 2017* https://www.gov.uk/government/publications/report-of-the-commission-on-human-medicines-expert-working-group-on-hormone-pregnancy-tests
Medicines and Healthcare products Regulatory Agency (MHRA) in collaboration with Bill and Melinda Gates Foundation	Project	Program of work: research to support the safer use of medicine during pregnancy, *September 2019* https://www.gov.uk/government/news/mhra-and-the-bill-melinda-gates-foundation-to-look-at-the-safer-effective-use-of-medicines-during-pregnancy
University of Birmingham and Birmingham Health Partners Centre for Regulatory Science and Innovation	Reports	Safe and effective medicines for use in pregnancy: a call to action Healthy Mum, Healthy Baby, Healthy Future: Report Sets out Vision to Deliver Safe, Effective and Accessible Medicines for use in Pregnancy https://www.birminghamhealthpartners.co.uk/wpcontent/uploads/2022/05/Final-Healthy-Mum-Healthy-Baby-Healthy-Future-Report-AW_Accessible-PDF-REDUCED-FILESIZE.pdf https://www.birmingham.ac.uk/documents/college-mds/centres/bctu/21560-policy-commission-maternal-health-report.pdf
Association of British Pharmaceutical Industry (ABPI)	Project	Maternal Health Project Group https://www.abpi.org.uk/our-ethics/patient-public-involvement/maternal-health-project-group-mhpg/
Second Wave Initiative, University of North Carolina Center for Bioethics	Project	https://bioethics.unc.edu/second-wave-initiative • Pregnancy and HIV/AIDS: seeking equitable study (PHASES) project: ending the evidence gap for pregnant women around HIV and co-infections: a call to action http://hivpregnancyethics.org/ • Zika and Beyond: pregnancy, research, and public health emergencies

### Approaches to Stimulate Investment in Therapeutics for Obstetric and Fetal Diseases

More complex proposals have been offered to stimulate investment in pregnancy therapeutics. This includes a call from the US Department of Health and Human Services (HHS) task force on research in pregnant women and lactating women (PRGLAC) and from the Duke-Margolis Center for Health Policy for Congress to grant FDA the authority to require sponsors to submit Obstetric Investigation Plans in early phases of research for investigational new drugs [8, 94]. Such a plan emulates Pediatric Investigational Plans or PIPs that are written during the development process of a new medicine to ensure that necessary data on the use of the medicine in children is obtained when it is safe to do so. While the implementation of PIPs has increased interest in many areas of pediatric drug development, it has not necessarily reduced the off-label use of drugs in children [95]. The Obstetric Investigational Plan approach fails to address the need to stimulate innovation for conditions specific to pregnancy and requires significant upfront collaborative work to identify the investigational programs not specific to pregnant populations that should be prioritized and/or compelled for study under the program. Such a proposal also does not address the requirements to generate non-clinical data and human safety data prior to inclusion of pregnant populations across ICH regions. Nor does it consider the resource impact (in particular on non-human primate studies) of requiring developmental and reproductive toxicity non-clinical studies in the earliest phases of development when the rate of attrition is higher than later in development. As discussed within the PRGLAC recommendations, this proposal should only be considered with a companion legal risk mitigation plan related to liability claims.

## Conclusion

Even before the COVID pandemic shone a light on the inequities of drug development for pregnant and lactating persons there was investment from agencies both public and private to address this issue. There is new guidance to facilitate scientific and ethical considerations for including pregnant and lactating persons in clinical trials, as well as new safety terminology to define and grade AEs in the mother, fetus and neonate. Progress is now being made in concert with patients and public to overcome the barriers to drug development and prescribing in pregnancy and lactation.
